# Comparative RNA-seq analysis reveals dys-regulation of major canonical pathways in ERG-inducible LNCaP cell progression model of prostate cancer

**DOI:** 10.18632/oncotarget.27019

**Published:** 2019-07-02

**Authors:** Parameet Kumar, Joyeeta Chakraborty, Gauthaman Sukumar, Clifton Dalgard, Raghunath Chatterjee, Roopa Biswas

**Affiliations:** ^1^ Department of Anatomy, Physiology and Genetics, Uniformed Services University of the Health Sciences, Bethesda, MD, USA; ^2^ Human Genetics Unit, Indian Statistical Institute, Kolkata, India; ^3^ Collaborative Health Initiative Research Program, Henry Jackson Foundation, Bethesda, MD, USA; ^4^ The American Genome Center, Uniformed Service University of the Health Sciences, Bethesda, MD, USA

**Keywords:** RNAseq, prostate cancer, LNCaP cells, ERG, mRNA

## Abstract

Prostate Cancer (CaP) is the second leading cause of cancer related death in USA. In human CaP, gene fusion between androgen responsive regulatory elements at the 5'-untranslated region of TMPRSS2 and ETS-related genes (ERG) is present in at least 50% of prostate tumors. Here we have investigated the unique cellular transcriptome associated with over-expression of ERG in ERG-inducible LNCaP cell model system of human CaP. Comprehensive transcriptome analyses reveal a distinct signature that distinguishes ERG dependent and independent CaP in LNCaP cells. Our data highlight a significant heterogeneity among the transcripts. Out of the 526 statistically significant differentially expressed genes, 232 genes are up-regulated and 294 genes are down-regulated in response to ERG. These ERG-associated genes are linked to several major cellular pathways, cell cycle regulation being the most significant. Consistently our data indicate that ERG plays a key role in modulating the expression of genes required for G1 to S phase transition, particularly those that affect cell cycle arrest at G1 phase. Moreover, cell cycle arrest in response to ERG appears to be promoted by induction of p21 in a p53 independent manner. These findings may provide new insights into mechanisms that promote growth and progression of CaP.

## INTRODUCTION

Prostate cancer (CaP) is the most commonly diagnosed male malignancy and a leading cause of cancer related deaths in USA [1–3]. Despite current advances in CaP research, there is a need for novel therapeutic targets for human CaP [4]. ERG is the most commonly overexpressed oncogene in CaP [5] and arises from a fusion between androgen receptor regulated promoter of TMPRSS2 and ETS-related genes (ERG) [6]. Various studies have reported that 50% of radical prostatectomy samples have a fusion of the TMPRSS2 with the coding sequences of ERG [7]. Subsequent studies established that the variability in the frequency of *TMPRSS2:ERG* fusion gene ranges from 27% to 79% [8]. Thus, there is a tremendous interest in dissecting the molecular mechanism by which the *TMPRSS2-ERG* fusion promote progression of CaP [9]. The discovery of the *TMPRSS2:ERG* gene fusion shifts the current paradigm in cancer genomics from experimental to bioinformatics approaches [7]. Here we report a unique cellular transcriptome associated with over-expression of ERG in ERG-inducible LNCaP cell model system of human CaP.

Over the decade a number of new cutting-edge technologies, including microarray-based transcriptomic analyses, have emerged as important tools for understanding the pathogenesis of CaP [10]. These technologies have added strongly to our understanding of the growth and development of human cancer [11], but have several major limitations. The recent advent of next-generation RNA sequencing (RNA-seq) technologies has overcome some of these limitations, and have thus created a whole new avenue for comprehensive transcriptome analysis [12]. RNA-seq is a powerful tool for studying gene expression and for analyzing changes in gene structure at the transcript level. Recently, RNA-seq has been increasingly used to explore and analyze the genetic factors of prostate cancers, such as fusion genes, somatic mutations, noncoding RNAs, alternative splicing events, and mutations in prostate cancer cell lines and tumors [13]. RNA-seq also has been used to dissect the factors involved in the conversion to androgen independence as well as radio-sensitization [14]. RNA-seq has led to the discovery of additional ETS fusion and has been used for analyzing novel genomic rearrangements to interrogate the whole cellular transcriptome [15].

To analyze the role of ERG over-expression in CaP development and progression, we performed genome-wide transcriptome profiling (RNA-seq) in LNCaP cell model system. Here we report the identification of novel differentially expressed genes (DEGs) associated with ERG over-expression in CaP. Our data suggest that the DEGs associated with key pathways are involved in cell cycle regulation. Our study demonstrates the role of ERG in reducing cell proliferation by modulating the expression of genes required for G1 to S phase transition, and thereby resulting in cell cycle arrest at G1 phase. We have also identified functionally important canonical pathways regulated by ERG, which may lead to novel therapeutic targets for ERG-associated CaP.

## RESULTS

### Effect of ERG on gene expression in LNCaP cells

**Figure 1:**
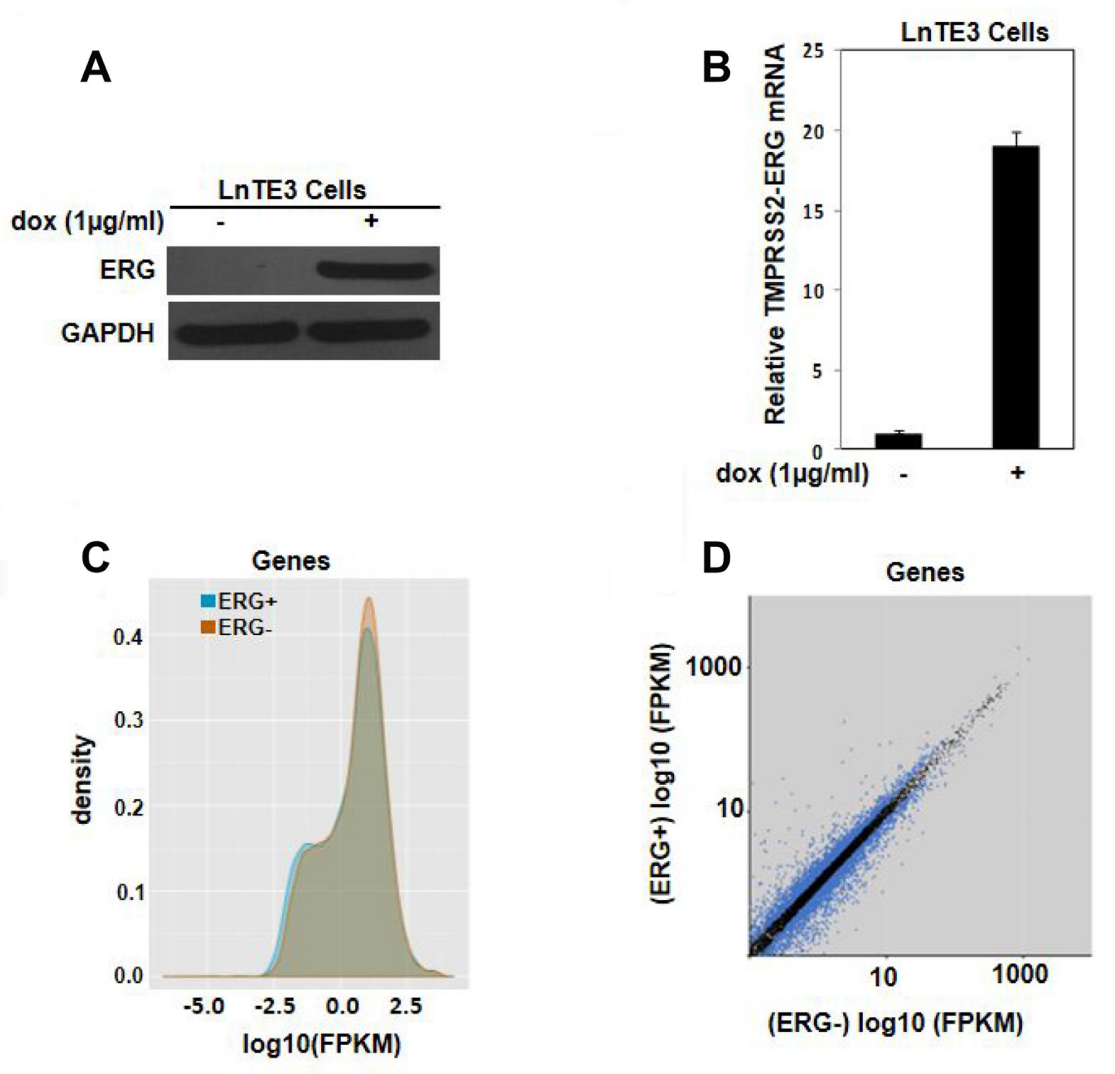
Transcriptomic analysis of ERG-inducible LNCaP cells. LnTE3 cells were treated with doxycycline (1 μg/ml) for 72 hours. ERG expression was analyzed by (**A**) immunoblot and (**B**) real-time PCR. The data is representative of three or more independent experiments. (**C**) The graph depicts the distribution and expression of all annotated genes (y-axis) and the intensity of their expression (x-axis as log10 (FPKM)) as obtained by global RNA-Seq analysis. (**D**) Scatter plot indicates the expression of significant genes (*q*-value < 0.05) in blue dots under the two experimental conditions, with the x-axis representing the FPKM values for ERG- and the y-axis representing the FPKM values for ERG+ samples.

To identify the gene signature associated with over-expression of ERG and to gain insight into the *TMPRSS2-ERG* gene fusion, we performed RNA-seq analysis. We employed tetracycline/doxycycline-mediated ERG-inducible LNCaP cell system designated as LnTE3 (LNCaP-lentivirus TMPRESS2:ERG3, inducible) cells [2, 16]. LnTE3 cells exhibits increased expression of ERG protein upon addition of doxycycline (Figure 1A) and a corresponding increase in expression of TMPRSS2-ERG mRNA (Figure 1B). LnTE3 cells that were not treated with doxycycline, and hence do not express ERG, served as a negative control. The total number of sequenced reads range from 16–23 million in ERG over-expressing cells (ERG+) and 10–22 million in ERG- LnTE3 cells (Supplementary Table 1). Approximately, 90% of the reads in each sample are aligned to the human genome (hg19).

Density plot showing the distribution of RNA-seq read counts (FPKM) of ERG- (orange area) and ERG+ (blue area) samples indicate that majority of the genes have similar distribution of RNA-seq read counts (grey area) (Figure 1C). Gene expression was determined by the number of reads uniquely mapped to the specific gene and the total number of uniquely mapped reads in the sample. Then fragments per kilobase of transcript per million mapped reads (FPKM), which takes into account both the gene length and sequencing depth on read count, was calculated. Figure 1D depicts the scatter plot of the transcripts with |Log_10_FC| ≥2 (*q*-value ≤ 0.05) in the ERG+ cells compared to ERG- cells. It is evident that ERG induces an alteration in gene expression profile in these LnTE3 cells.

We have identified a total of 526 statistically significant DEGs in ERG+ cells compared to ERG- LnTE3 cells (Supplementary Data 1). Approximately 44% (232) of the differentially expressed genes are up regulated, while 56% (294) of the DEGs are down regulated in ERG+ LnTE3 cells compared to the ERG- control cells. Hierarchical clustering of 526 DEGs indicated two distinct clusters for ERG+ and ERG- LnTE3 cells (Supplementary Figure 1).

**Figure 2:**
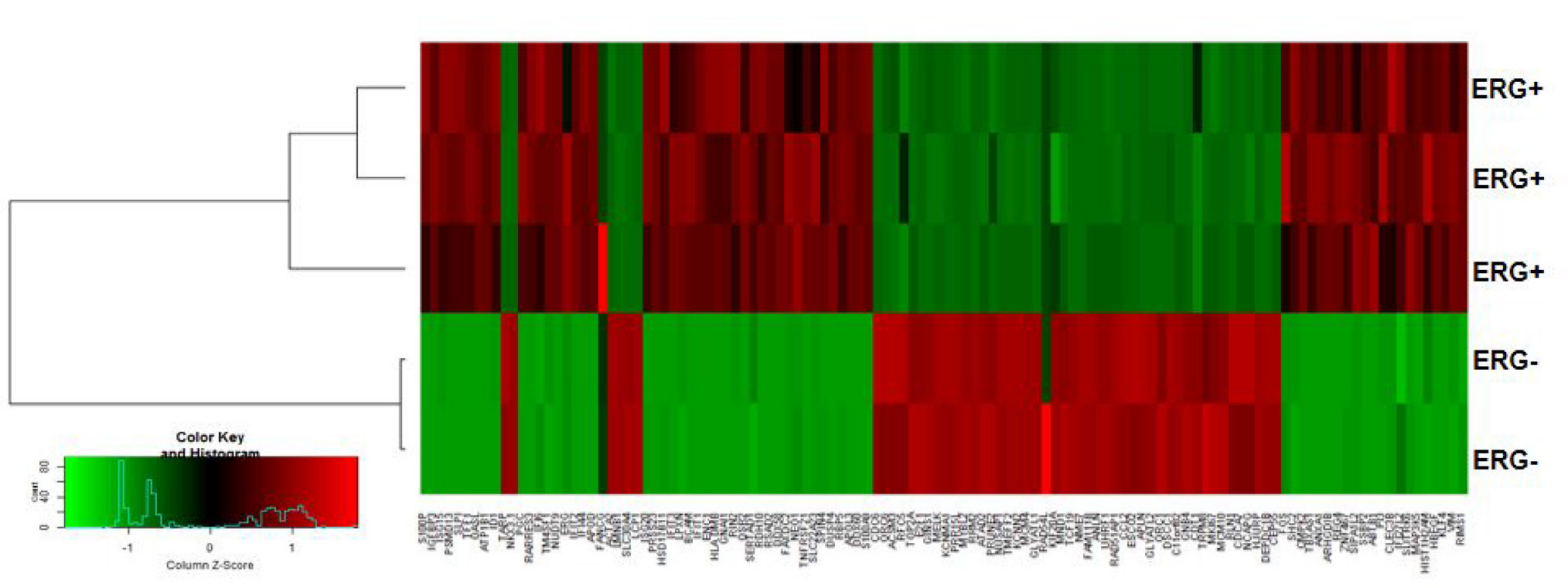
ERG-associated transcripts in CaP cells. Hierarchical clustering of transcripts significantly altered in expression can distinguish between ERG+ and ERG– LnTE3 cells. The heat map indicates the expression level of the transcripts significantly altered in ERG+ compared to ERG– LnTE3 cells: red represents increased expression, while green represents reduced expression. The expression levels are continuously mapped on the color scale provided at the bottom of the figure.

For further downstream analysis, we considered a set of 117 DEGs with |Log_2_FC| ≥2 in ERG+ compared to ERG- LnTE3 cells. As depicted in Figure 2, hierarchical clustering of these 117 genes include a total of 7 clusters, among which 5 clusters are dominant. Z score was calculated for each of the 117 genes. The top genes that are induced by ERG include *TFF1*, *RSAD2*, *OASL*, *IFIT2*, *IFIT1*, *S100P*, *IFIT2*, *REG4*, *RARRES3*, *IFIT3*, *ARHGDIB*, *ANXA1*, PRSS23, *IGFBP3*, *APOL3*, *FOS* and *S100A9*. While those genes that are suppressed by over-expression of ERG include *APLN*, *CCL2*, *SLC30A4*, *LCP1*, *GLYATL2*, *FAM111B*, *TARP*, *RLN1*, *ESCO2* and *TRPM8* (Supplementary Data 1).

### Functional analyses of differentially expressed genes

Next we performed *in silico* analyses of the significant DEGs in ERG+ LnTE3 cells compared to ERG- control cells (≥ 2.0 fold change cut-off; *q*-value ≤ 0.05) (*n =* 526; Supplementary Data 1) using Ingenuity Pathway Analysis (IPA). Table 1 summarizes the ERG-induced top five diseases and disorders; and include Cancer (*p*-value range = 1.20E-04–4.96E-27), Organismal injury and abnormalities (*p*-value range=1.20E-04 –4.96E-27), Reproductive system disease (*p*-value range=1.20E-04–4.96E-27), Respiratory disease (*p*-value range=9.96E-05–1.33E-16), Gastrointestinal disease (*p*-value range=1.16E-04–1.25E-13). The top ranked bio-functions significantly affected by ERG over-expression include Cell Cycle (*p*-value range=1.42E-04–3.98E-33, z-score = –0.947), Cellular Growth and Proliferation (*p*-value range = 1.23E-04–1.68E-31, z-score = –3.881), Cellular Development (*p*-value range = 1.23E-04–3.73E-27, z-score = –3.463), Cell Death and Survival (*p*-value range = 1.37E-04–3.91E-27, z-score = –2.125), and Cellular Assembly and Organization (*p*-value range = 1.42E-04– 4.46E-24, z-score = –0.378).

**Table 1:** Top diseases and bio-functions enriched by differentially expressed genes with increased expression of ERG in LnTE3 cells

Top diseases		
Diseases and Disorders	*p* value range	Molecules
Cancer	1.20E-04–4.96E-27	449
Organismal Injury and Abnormalities	1.20E-04–4.96E-27	449
Reproductive System Disease	1.20E-04–4.96E-27	264
Respiratory Disease	9.96E-05–1.33E-16	95
Gastrointestinal Disease	1.16E-04–1.25E-13	345

Top Bio functions			
Molecular and Cellular Functions	*p* value range	z-score	Molecules
Cell Cycle	1.42E-04–3.98E-33	–0.947	171
Cellular Growth and Proliferation	1.23E-04–1.68E-31	–3.881	259
Cellular Development	1.23E-04–3.73E-27	–3.463	219
Cell Death and Survival	1.37E-04–3.91E-27	–2.125	225
Cellular Assembly and Organization	1.42E-04–4.46E-24	–0.378	127

Top five enriched disease and biological functions as analyzed with Ingenuity Pathway Analysis software in the experimental dataset. z-score, measure of predicted changes, increased (positive z-score) or decreased (negative z-score). Molecules, number of genes in the dataset, which are represented in the top disease or Bio-functions.

**Figure 3:**
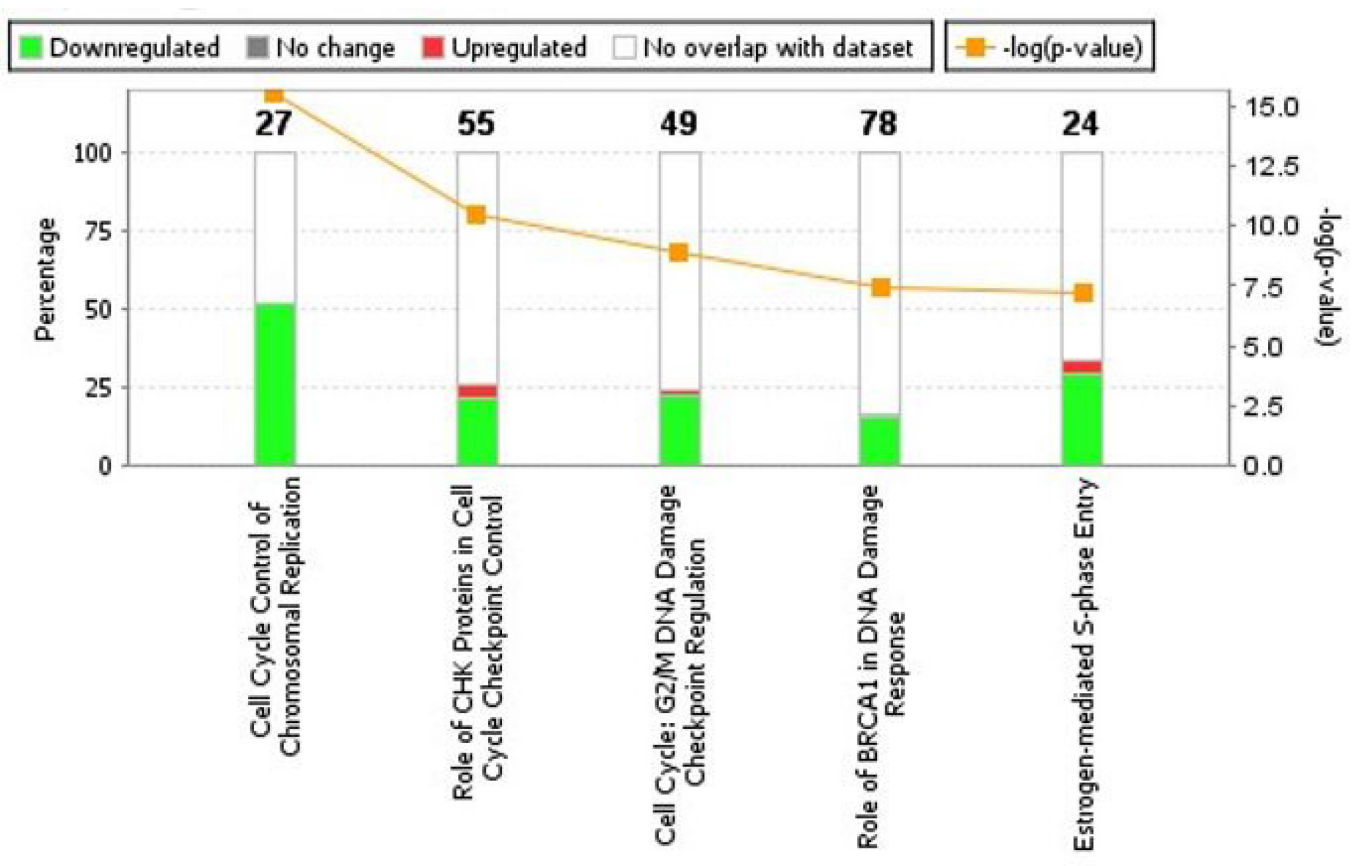
Analyses of canonical pathways in ERG-inducible LnTE3 cells. Top five canonical pathways enriched by DEGs (differentially expressed genes) are depicted. Canonical pathways significantly altered by increased ERG expression were generated by IPA. The orange line represents a ratio of regulated proteins to all proteins in the pathway. The stacked bar chart for each canonical pathway displays the number of genes that were significantly up-regulated (red), and down-regulated (green). The molecules/genes in a given pathway that were not found in our list of significantly regulated genes are termed unchanged (grey) or not overlapping with our dataset (white). The numerical value at the top of each bar represents the total number of genes/molecules in the canonical pathway.

Subsequent to analyses of cellular processes affected by ERG expression, we analyzed canonical pathways enriched with ERG over-expression. The top-five statistically significant canonical pathways affected by increased expression of ERG include, Cell Cycle control of chromosomal replication (*p*-value = 2.69E-16, z-score = NaN), Role of CHK proteins in Cell Cycle checkpoint control (*p*-value = 3.16E-11, z-score = 0.707), Cell Cycle: G2/M DNA damage checkpoint regulation (*p*-value = 1.34E-09, z-score = 1.508), Role of BRCA1 in DNA damage response (*p*-value = 4.05E-08, z-score = –1.0) and Estrogen-mediated S-phase entry (*p*-value = 5.51E-08, z-score = –2.82) (Figure 3, Table 2).

**Table 2:** Top canonical pathways enriched by differentially expressed genes obtained with increased expression of ERG in LnTE3 cells

Top canonical pathways			
Pathways	*p* value	z-score	Overlap, ratio
Cell Cycle Control of Chromosomal Replication	2.69E-16	NaN	51.9% (14/27)
Role of CHK Proteins in Cell Cycle Checkpoint Control	3.16E-11	0.707	25.5% (14/55)
Cell Cycle: G2/M DNA Damage Checkpoint Regulation	1.34E-09	1.508	24.5% (12/49)
Role of BRCA1 in DNA Damage Response	4.05E-08	–1.0	16.7% (13/78)
Estrogen-mediated S-phase Entry	5.51E-08	–2.82	33.3% (8/24)

Significantly enriched canonical pathways in the experimental dataset with ERG induction in LnTE3 cells are shown. z-score; is a measure of predicted change (activated or reduced) of the pathways. NaN, not a number. Overlap, ratio; percentage of genes in the dataset, as represented in the pathway. Numbers in brackets show number of gene in the data set to the total number of genes in the pathway in the reference gene set.

**Figure 4:**
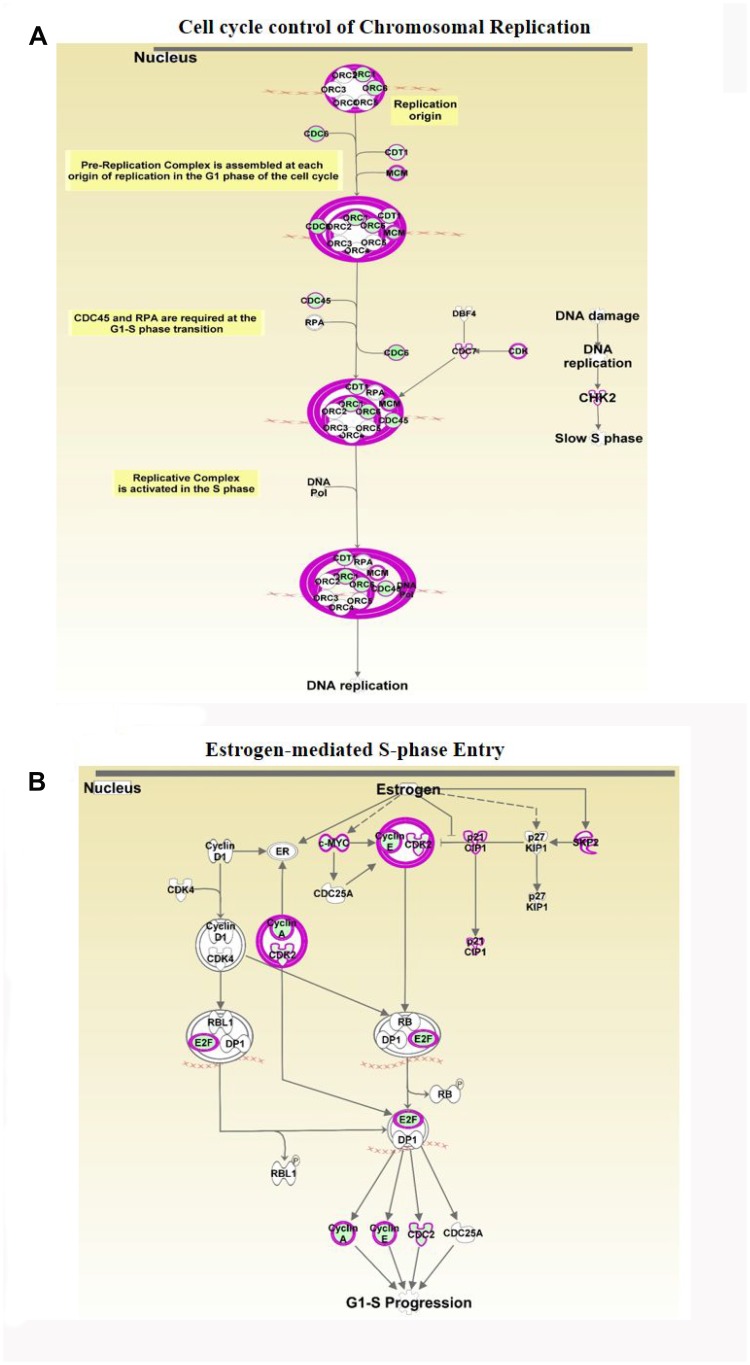
Analyses of ERG-associated cellular pathways. Differentially expressed genes obtained by RNA-seq in the ERG-inducible LnTE3 cells were analyzed using IPA. Canonical pathway analysis revealed several significantly deregulated pathways including: (**A**) Cell Cycle Control of Chromosomal Replication and (**B**) Estrogen-Mediated S-phase Entry. Majority of the focus molecules are present in the differentially expressed genes. Significantly up-regulated gene are indicated in red and down-regulated genes are in green, and those present within our data set but not significant are shown in grey. Arrows indicate gene products which were found to be oppositely regulated.

**Figure 5:**
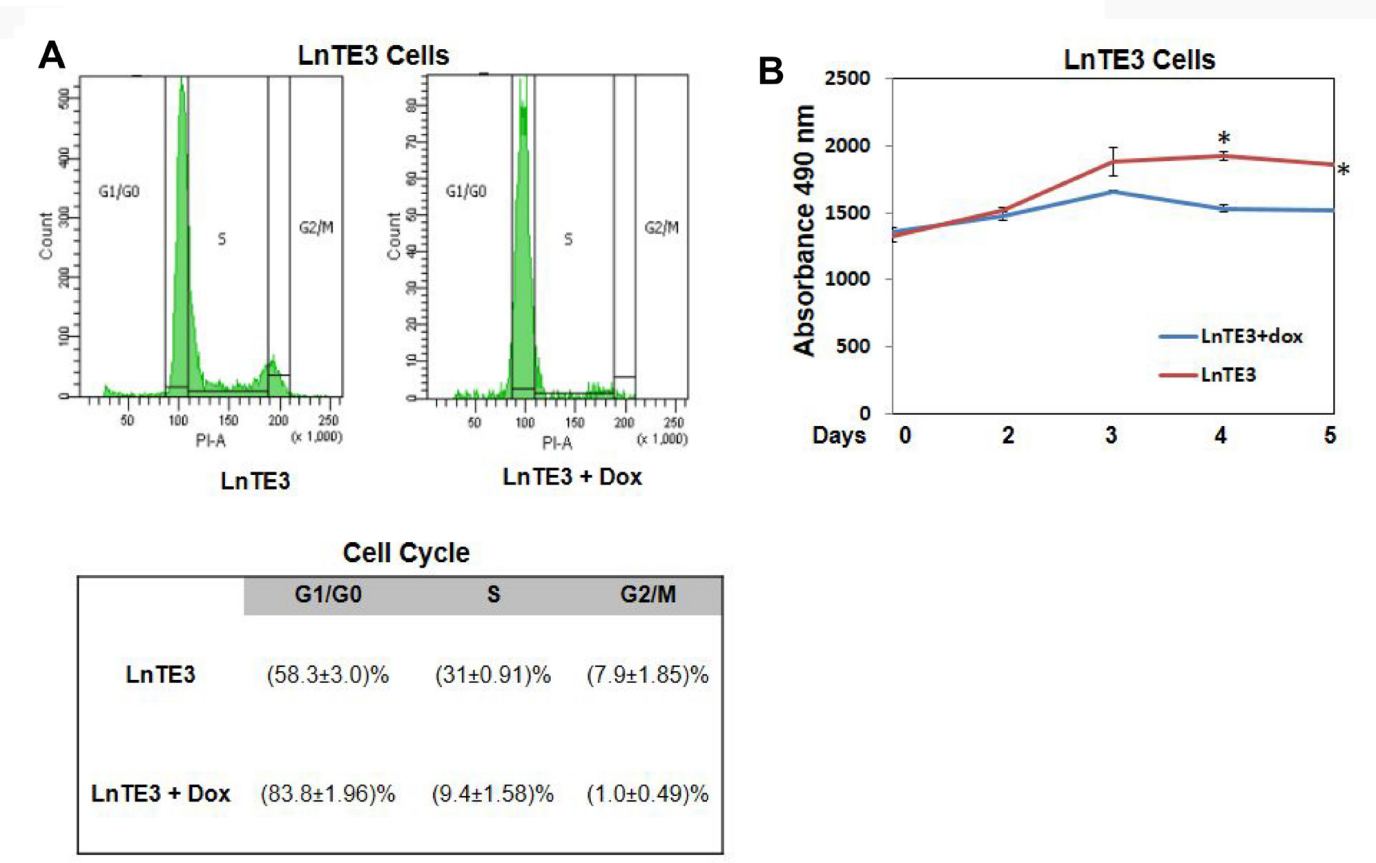
Analyses of cell cycle in ERG-inducible CaP cells. (**A**) LnTE3 cells were treated with or without doxycycline (1 μg/ml) for 72 hr, cells were synchronized by serum deprivation, and cell cycle distribution was analyzed with the help of BD LSR II flow cytometer. The data (mean ± SD of three experiments) indicate the relative percentage of cells at G0/G1, S, and G2/M phase of the cell cycle. (**B**) Cell proliferation assay was performed by measuring cell number over a 2 to 5 day time course. The reported results are the mean of three independent experiments (*p* < 0.05).

Cell cycle control of chromosomal replication was observed as the top canonical pathway affected by ERG over-expression and indicate slow S phase in response to DNA damage. Our data also illustrate that the 14 genes (*ORC6*, *ORC1*, *MCM7*, *MCM6*, *MCM5*, *MCM4*, *MCM3*, *MCM2*, *CHEK2*, *CDT1*, *CDK2*, *CDC45*, *CDC7* and *CDC6*) involved in this cellular process are all significantly down-regulated by ERG (Figure 4A, Table 2). Estrogen-mediated S-phase entry was also amongst the top canonical pathways found to be enriched in ERG+ LnTE3 compared to ERG- control cells (Figure 4B, Table 2). As shown in Figure 4B, increased expression of ERG suppresses the expression of *c-MYC*, *E2F*, *SKP2*, *CDK2*, *CDC2* as well as *cyclin A* and *cylcin E*. Moreover, we find that ERG induction also induces p21 expression (also known as CDKN1A or p21^WAF1/CIP1^). Since ERG modulates the expression of majority of the genes involved in cell cycle regulation (Table 2, Figure 3, Figure 4A and 4B) we performed cell cycle progression studies in LnTE3 cells. LnTE3 cells were treated with dox (1 μg/ml) to induce ERG and synchronized by serum deprivation. We observe that 24 h after synchronization, the fraction of cells in the S-phase was reduced (from 31% to 9%) in ERG+ LnTE3 cells as compared to control ERG- LnTE3 cells (Figure 5A), indicating that over-expression of ERG results in a slower cell cycle progression. We further performed proliferation assays over a 2 to 5 day time course. As depicted in Figure 5B we find that high ERG significantly reduces proliferation of LnTE3 cells. Collectively, our data indicate that ERG plays a key role in modulating the expression of genes required for G1 to S phase transition, resulting in the cell cycle arrest at G1 phase in LnTE3 cells (Figure 5A).

### Gene networks affected by ERG over-expression

**Figure 6:**
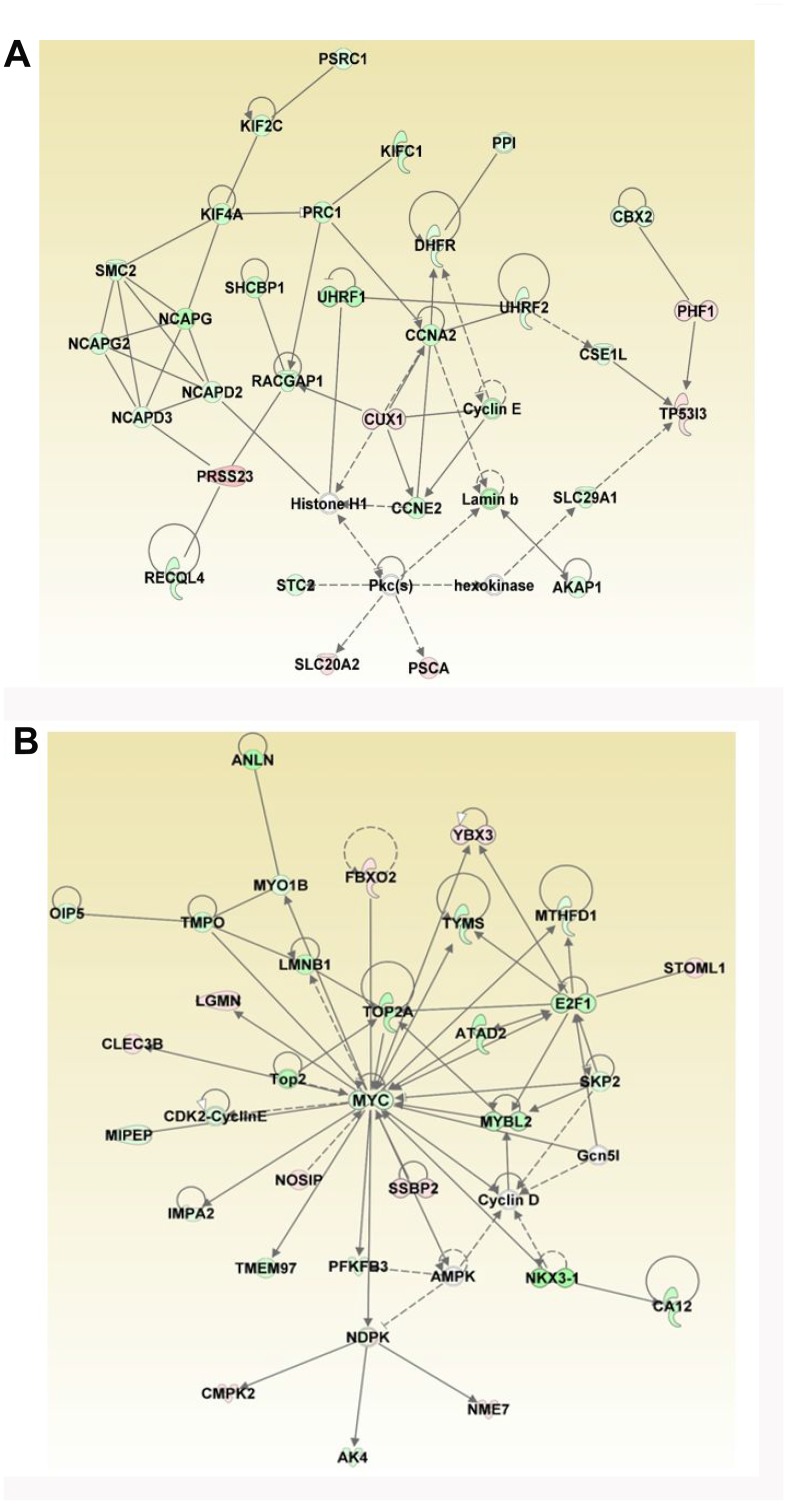
Analyses of ERG-associated networks. Top two gene networks generated by IPA analysis include (**A**) Cellular Assembly and Organization, DNA Replication, Recombination, and Repair, Cell Cycle and (**B**) Cell Cycle, Hematological System Development and Function, Hematopoiesis. Majority of the focus molecules are present in differentially expressed genes. Solid arrows represent the genes that interact directly, dotted arrows represent indirect interactions between genes. Network I consists of six down regulated and eight up-regulated genes and Network II consists of five down regulated and eight up-regulated genes.

The DEGs were further analyzed for regulatory biological relationships mediated by the ERG over-expression. Table 3 lists the top five gene networks with highest score and focus molecules associated with over-expression of ERG. The top two major networks include 29 focus molecules each (Table 3, Figure 6A and 6B). The roles and diseases related to Network I are cellular assembly and organization, DNA replication, recombination, and repair, Cell cycle and those related to Network II are Cell cycle, Hematological system development and function, Hematopoiesis (Figure 6A and 6B). In Network I, the genes that are up-regulated include *PRSS23*, *CUX1*, *PHF1*, *TP53I3*, *PSCA* and *SLC20A2* (shown in the red). Moreover, the different Cyclins (CCNA2, CCNE2 and Cyclin E) which play a role in cell cycle G1/S transition are down-regulated in response to ERG as illustrated in Network I. Network II reveals *MYC* as one of the focus molecules. The key genes that are down regulated by ERG include *MYC*, *NKX3-1*, *MYBL2*, *TOP2A* and *E2F1*. Those genes that are up-regulated by ERG induction include *LGMN*, *FBXO2*, *NOSIP*, *SSBP2*, *YBX3*, *STOML1*, *NME7*, *CMPK2* and *CLEC3B*.

**Table 3:** Characteristic variation of differentially expressed genes

Score	Focus molecules	Representative differentially expressed genes in ERG+ LnTE3 cells	Genes function and description
44	29	AKAP1**↓**, CBX2**↓**, CCNA2**↓**, CCNE2**↓**, CSE1L**↓**, CUX1**↑**, Cyclin E, DHFR**↓**, hexokinase, Histone H1, KIF2C**↓**, KIF4A**↓**, KIFC1**↓**, Lamin b, NCAPD2**↓**, NCAPD3**↓**, NCAPG**↓**, NCAPG2**↓**, PHF1**↑**, Pkc(s), PPI, PRC1**↓**, PRSS23**↑**, PSCA**↑**, PSRC1**↓**, RACGAP1**↓**, RECQL4**↓**, SHCBP1**↓**, SLC20A2**↑**, SLC29A1**↓**, SMC2**↓**, STC2**↓**, TP53I3**↑**, UHRF1**↓**, UHRF2**↓**	Cellular Assembly and Organization, DNA Replication, Recombination, and Repair, Cell Cycle
44	29	AK4**↓**, AMPK, ANLN**↓**, ATAD2**↓**, CA12**↓**, CDK2-CyclinE, CLEC3B**↑**, CMPK2**↑**, Cyclin D, E2F1**↓**, FBXO2**↑**, Gcn5I, IMPA2**↓**, LGMN**↑**, LMNB1**↓**, MIPEP**↓**, MTHFD1**↓**, MYBL2**↓**, MYC**↓**, MYO1B**↓**, NDPK, NKX3-1**↓**, NME7**↑**, NOSIP**↑**, OIP5**↓**, PFKFB3**↓**, SKP2**↓**, SSBP2**↑**, STOML1**↑**, TMEM97**↓**, TMPO**↓**, Top2, TOP2A**↓**, TYMS**↓**, YBX3**↑**	Cell Cycle, Hematological System Development and Function, Hematopoiesis
40	27	APC (complex), APC-CDC20, AURKB**↓**, BUB1**↓**, BUB1B**↓**, CCNB2**↓**, CDC20**↓**, CDCA5**↓**, Cdk, CENPH**↓**, CENPK**↓**, CENPM**↓**, CENPU**↓**, CKS2**↓**, CKS1B**↓**, Cyclin B**↓**, DLGAP5**↓**, ELK4**↓**, ERK, FBXO5**↓**, INCENP**↓**, KIF20A**↓**, MAD2L1**↓**, NDC80**↓**, NUF2**↓**, NUSAP1**↓**, PKMYT1**↓**, Plk, PTTG1**↓**, RNA polymerase I, Scf Trcp beta, SPC24**↓**, TPX2**↓**, ZFP36L1**↑**, ZWINT**↓**	Cell Cycle, Cellular Assembly and Organization, DNA Replication, Recombination, and Repair
40	27	7S NGF, Beta Tubulin, DEPDC1B**↓**, DSCC1**↓**, FANCG**↓**, FANCI**↓**, FEN1**↓**, FOS**↑**, GST, HES6**↓**, HMMR**↓**, KIAA0101**↓**, KLK3**↓**, LNX2**↑**, MAFB**↓**, MDC1**↓**, Mir122ab, MutS alpha, OPTN**↑**, PCNA**↓**, POLD4**↑**, PRIM1**↓**, Rab11, RAD54L**↓**, Rfc, RFC2**↓**, RFC3**↓**, RFC4**↓**, RFC5**↓**, SULT2B1**↓**, TCF, TLE1**↑**, UBE2T**↓**, XRCC3**↓**, ZNF467**↑**	DNA Replication, Recombination, and Repair, Cancer, Organismal Injury and Abnormalities
33	24	ASF1B**↓**, ATM/ATR, Caspase 3/7, Cdc2, CDC6**↓**, CDC7**↓**, CDC45**↓**, CDT1**↓**, CHEK1**↓**, Cyclin A, DHCR24**↓**, E2f, FANCD2**↓**, FKBP5**↓; **, GINS1**↓**, GINS2**↓**, GINS3**↓**, GMNN**↓**, Jnk, MAP1LC3, Mcm, MCM2**↓**, MCM3**↓**, MCM4**↓**, MCM5**↓**, MCM6**↓**, MCM7**↓**, MCM8**↓**, MCM10**↓**, ORC1**↓**, ORC6**↓**, Rb, RPA, RRM2**↓**, TH2 Cytokine	DNA Replication, Recombination, and Repair, Connective Tissue Disorders, Developmental Disorder

Notes: **↑** upregulation. **↓**downregulation.

In the third network, there are 27 focus molecules involved in Cell cycle, Cellular assembly and organization, DNA replication, recombination, and repair network. Majority of the genes involved in this network are suppressed by ERG-induction except *ZFP36L1* (Table 3). ERG also modulates the expression of *FOS* and *PCNA*, which are focus molecules in Network IV that includes DNA replication, recombination, and repair, cancer, Organismal injury and abnormalities (Table 3). The fifth network includes DNA replication, recombination, and repair, Connective tissue disorders, and Developmental disorder. Interestingly, majority of the genes involved in these networks are down-regulated in ERG+ cells compared to ERG- LnTE3 cells.

### Expression and validation of DEGs

**Figure 7:**
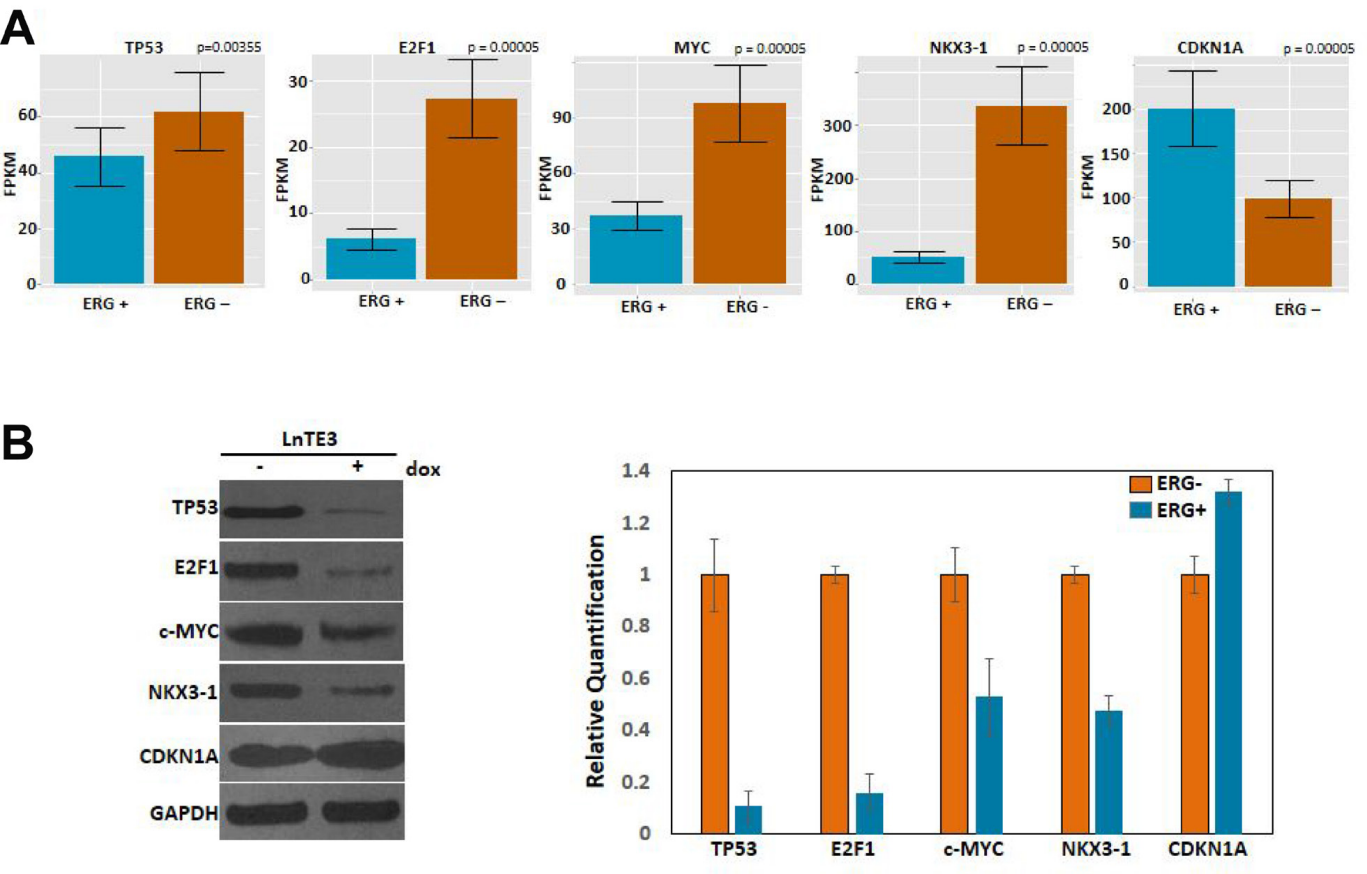
Expression and validation of DEGs. (**A**) The bar plots represent expression of genes, including TP53, E2F1, c-MYC, NKX3-1 and *CDKN1A*, in ERG+ as compared to ERG- LnTE3 cells, measured in FPKM. Each gene and transcript expression value is annotated with error bars. (**B**) Immunoblot analyses of these genes were performed in ERG+ and ERG– LnTE3 cells. Adjacent graph depicts the protein quantification using ImageJ software. The data includes mean and standard deviation from at least three independent experiments.

Our RNA-seq data indicate that TP53, CDKN1A and E2F1, are the top molecules from upstream regulator analyses (generated by IPA). CDKN1A, E2F1 and c-MYC were also significantly enriched in one of the top canonical pathways “estrogen-mediated S-phase entry” (see Figure 4B). Moreover, E2F1, c-MYC and NKX3-1 appeared as major focus molecules of Network II (see Figure 5B). ERG induction in LnTE3 cells significantly alters the expression of TP53, CDKN1A, E2F1, c-Myc and NKX3.1 genes. While c-MYC, TP53, E2F1 and NKX3-1 were suppressed by ERG induction in LnTE3 cells, *CDKN1A* was up-regulated (Figure 7A). Validation of the expression of these genes was further performed by immunoblot analyses. As shown in Figure 7B, protein expression data exhibits a trend that is consistent with that obtained from RNA-seq.

### GO term analysis of differentially expressed genes

**Figure 8:**
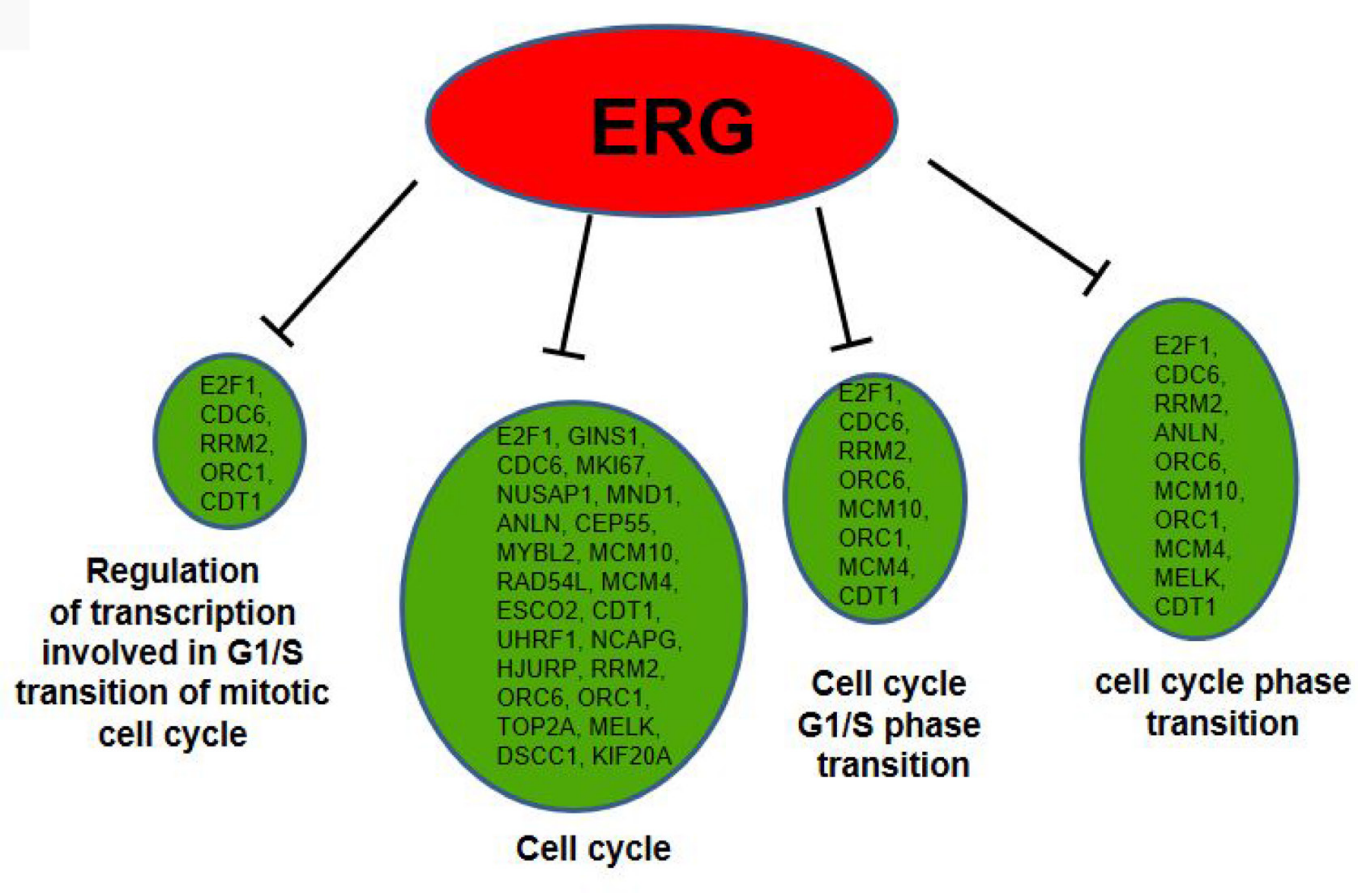
GO term analysis for differentially expressed genes. GO analyses indicate many ERG modulated genes to be associated with regulation of cell cycle, Cell cycle G1/S phase transition, Regulation of transcription involved in G1/S transition of mitotic cell cycle and cell cycle transition (red color represents up-regulated and green color represents down-regulated genes).

To determine the proportion of input genes in ERG+ LnTE3 cells involved in a particular cellular process or function compared to that in ERG- control cells, we performed Gene Ontology (GO) analysis of the DEGs present in the 5 dominant clusters (described in Figure 2). GO enrichment analysis (FDR<0.1 and Fold Enrichment ≥2), identified many processes and functions that are regulated by ERG, including regulation of cell cycle (FDR = 2.53E-10), Cell cycle G1/S phase transition (FDR = 0.002663973), Regulation of transcription involved in G1/S transition of mitotic cell cycle (FDR = 0.000780178), and cell cycle phase transition (FDR = 0.007444829) (Figure 8).

## DISCUSSION

Prostate cancer is a multifactorial disease caused by a series of genetic alterations [17]. The TMPRSS2:ERG gene fusion is detected in 50% of the CaP patients [18]. To investigate the characteristics of ERG-dependent and ERG-independent prostate cancer, RNA from these two groups was subjected to RNA sequencing. We identified a total of 526 differentially expressed genes that are significantly altered by increased expression of ERG in LNCaP cells. These differentially expressed genes are associated with many pathways and functions. Our data suggest that the most significant effect is on cell cycle regulation. Consistently, we also observe enrichment of major cell cycle-related canonical pathways with increased expression of ERG in CaP cells.

The top genes that are elevated with over-expression of ERG and are known to be regulators of cancer phenotype include TFF1, S100P, REG4, ARHGDIB, ANXA1, PRSS23, IGFBP3, APOL3, FOS and S100A9. *TFF1* (Trefoil factor-1) also known as *pS2* [19], is the most up-regulated gene induced by ERG. This gene belongs to the family of trefoil factors, that are classical estrogen-regulated genes [20] and is overexpressed in several types of cancers including prostate cancer [21, 22]. TFF1 enhances cell migration and invasion [23] and has been shown to be a marker of hormone responsiveness in tumors [24]. Previous reports indicate that patients with advanced prostate cancer have significantly higher plasma concentrations of TFF1 [25]. High S100P expression is observed in several types of cancers and has been shown to mediate tumor growth, drug resistance, and metastasis [26]. Additionally, S100P is regulated by androgen [27], and high S100P promotes prostate cancer progression [28]. Consistent with previous studies [29], our data also indicate that ERG induces the expression of S100P. We also detected high expression of REG4 in ERG + compared to ERG- LnTE3 cells. REG4 has been shown to be a prognostic factor in clinically localized prostate cancer [30] and a promising marker of hormone refractory metastatic prostate cancer [31]. REG4 has been shown to enhance metastasis in gastric carcinomas [32] and also contributes to invasiveness in pancreatic [33] and colorectal carcinoma [34]. ARHGDIB also known as RhoGDI2 has been identified as a proto-oncogene and is up regulated in multiple human cancer [35, 36]. RhoGDI2 also regulates epithelial-mesenchymal transition, which is responsible for invasiveness during tumor progression [37]. Annexin A1 (ANXA1) is overexpressed in the invasive stages of prostate cancer [38] and is involved in the acquisition and maintenance of stem-like/aggressive features in prostate cancer [39]. Serine protease PRSS23 is known to be associated with tumor progression in various types of cancers and is co-expressed with estrogen receptor α (ERα) [40]. IGFBP3 levels are significantly elevated in prostate cancer patients urine [41] and is consistent with our data. Moreover, a case-control study has shown the association between a SNP within the APOL3 locus and prostate cancer risk [42].

The genes that are suppressed by over-expression of ERG in LnTE3 cells includes *APLN*, *CCL2*, *SLC30A4*, LCP1, *GLYATL2*, *FAM111B*, *TARP*, *RLN1*, *ESCO2* and *TRPM8*. Our data indicate that *GLYATL2*, an ETV1 target gene [43, 44], is reduced with ERG over-expression in CaP cells. *FAM111B* common variants are associated with prostate cancer susceptibility in the Japanese population [45]. *TRPM8* variant α is generally overexpressed in prostate cancer [46] but contrary to this our data show that it is suppressed in ERG over-expressing LnTE3 cells. RLN1 is known to form a fusion with RLN2 in LNCaP cells as well as in normal and prostate cancer tissues [47]. We find that ERG causes reduced expression of RLN1. SLC30A4, another gene whose expression is suppressed by ERG, a zinc transporter (ZnT4), has been shown to promote the progression of CaP from early prostate disease to invasive prostate cancer [48].

Disruption of various signaling pathways is a characteristic feature of tumors [49, 50]. Our data illustrate the enrichment of key cellular signaling pathways involved in the carcinogenic process. The top canonical pathways altered with ERG over-expression are mainly associated with Cell cycle control. (Table 1). The top upstream regulators that emerge from IPA analyses include TP53, CDKN1A, E2F1 and CCND1 molecules. The precise switch from G1 to S phase is vital for cell proliferation and its mis-regulation promotes oncogenesis [51]. We find that ERG suppresses the expression of 51.9% of the genes involved in cell cycle control of chromosomal replication, including origin recognition complex (ORC1 and ORC4) as well as initiation factors, including CDT1, CDC6 and Mcm, essential for the assembly of the pre-replication complex. ORC-depleted cells have been shown to be arrested in G1 phase [52]. Moreover, it has been established that deregulation of CDC6 expression poses a serious risk of carcinogenesis and its down-regulation inhibits cell proliferation and promotes apoptosis [53]. CHK is required for checkpoint mediated cell cycle arrest in response to DNA damage, and suppression of CHK1 by ETS family members has been shown to promote DNA damage response [54]. Consistently, our data indicate that CHK is suppressed by ERG expression in LnTE3 cells. Cdc45, an essential protein required for the initiation of DNA replication, is also suppressed by increased expression of ERG in LnTE3 cells. This is in concurrence with a previous study which demonstrated that increased expression of ERG leads to genomic instability [55].

The transcription factor E2F1 is active during G1 to S transition and is involved in cell cycle progression. Here we also report the reduced expression of E2F1 with increased expression of ERG in LnTE3 cells. Further analyses also indicate that ERG causes slow G1 to S phase transition in ERG+ LnTE3 cells. These findings are consistent with a recent study, which demonstrated that increased ERG expression causes reduced proliferation and accumulation of cells in G1 phase [56]. p21^WAF1/CIP1^ associates directly with E2F1 and suppresses its transcriptional activity [57]. Our RNA-seq and immunoblot data demonstrate that ERG promotes increased expression of p21^WAF1/CIP1^ in ERG+ LnTE3 cells. p21^WAF1/CIP1^ is a potent inhibitor of CDK activity and can suppress cell growth and proliferation by blocking cell cycle progression in the G1/S phase transition [58]. Moreover, p21 over-expression has been associated with severe clinical outcome with androgen deprivation therapy in prostate cancer [59, 60]. Elevated levels of p21 also appears to be associated with invasive phenotype of cancer [28]. Increased p21 expression has been observed in cervical carcinoma, brains tumors and is associated with recurrence and metastasis of ovarian cancer [61–63]. Furthermore, p21 is induced by both p53-dependent and independent mechanisms in response to DNA-damaging agents and is known to induce apoptosis [2, 64, 65]. Since our earlier studies have demonstrated the reduced expression of p53 with increased levels of ERG in CaP cells [2], it appears that in these ERG-inducible LnTE3 cells, p21 expression can be regulated independent of p53. This phenomenon is consistent with previous reports [57, 66]. The up-regulation of p21 expression promoted by increased levels of ERG is clearly important for understanding p53-independent growth arrest. The regulation of p21 by factors like ERG suggests that it is a more universal cell cycle regulator, as in the present study p21 expression is clearly regulated independent of p53. Moreover, it has been demonstrated that p21 binding to PCNA causes G1 and G2 cell cycle arrest in p53-deficient cells [67]. Thus, ERG appears to play a critical role in p21 induction following DNA damage and is perhaps protecting cells from apoptosis by suppressing p53.

It is well established that increased expression of Myc induces cell cycle progression and its down-regulation impairs cell cycle progression [68]. Myc is suggested to play an important role in the transition from quiescence state to proliferation [69]. It has been shown that Myc disrupts the PCNA-p21 interaction, thus refining p21-dependent inhibition of PCNA and DNA synthesis [57]. Here we report that ERG reduces the expression of PCNA and Myc in LnTE3 cells. However, this is contrary to that observed in ERG-positive VCaP cell lines, which have increased Myc expression [70]. Individual cancer cell lines provide a stage of the cancer at the time the biopsy was taken [71]. This variability may be due to the differences in cancer stages in these two different cell lines.

In summary, we observe the enrichment of major canonical pathways with ERG induction in LnTE3 cells. Our data suggest that, the differentially expressed genes in key pathways are associated with cell cycle regulation. Moreover, ERG suppresses ~50% of the genes required for cell cycle control of chromosomal replication in LnTE3 cells. Thus, the RNA-seq data and cell cycle analyses collectively indicate that ERG plays a key role in modulating the expression of genes required for G1 to S phase transition, resulting in cell cycle arrest at G1 phase. This seems to be favored by induction of the key cell cycle regulated gene p21^WAF1/CIP1^. Moreover, the induction of p21^WAF1/CIP1^ by ERG appears to be independent of p53. Our present data, clearly suggests the role of ERG in reducing proliferation by slowing down G1 to S phase transition in this LNCaP cell model system.

## MATERIALS AND METHODS

### Cell cultures and antibodies

LNCaP cell line was transduced with an inducible lentiviral ERG construct (LNCaP-lentivirus TMPRESS2:ERG3 inducible) to establish stable doxycycline-inducible ERG expressing LnTE3 cell line [2, 16]. The cell lines were cultured in RPMI 1640, supplemented with 10% Tet System Approved Fetal Bovine Serum (Clontech Laboratories, Inc. Mountain View, CA, USA) and puromycin (Sigma, St. Louis, MO, USA) with or without doxycycline (Dox, 1 μg/ml) as per requirements and characterized as described [2, 16]. Antibodies used were as follows: anti-GAPDH (Millipore MAB374), anti-ERG (Abcam ab92513), anti- p21Waf1/Cip1, anti-E2F1 and anti- c-Myc Antibody (Cell Signaling 2946, 3742 and 9402, respectively), anti-p53 DO1 (Santa Cruz biotech, sc126), and anti-NKX3.1 (Biocare Medical SKU 422).

### Transcriptome profiling by RNA sequencing

Total RNA was quantified via a fluorescence dye-based methodology (RiboGreen) on a Spectramax Gemini XPS plate reader (Molecular Devices, Mountain View, CA, USA). RNA integrity was assessed using gel-based electrophoresis on an Experion Automated Electrophoresis System (Bio-Rad, Hercules, CA, USA). All samples used as input for library preparation were RQI > 9.0. Total RNA input of 200 ng was used for library preparation using the TruSeq Stranded mRNA Library Preparation Kit (Illumina, San Diego, CA, USA). Sequencing libraries were quantified by PCR using KAPA Library Quantification Kit for NGS (Kapa, Wilmington, MA, USA) and assessed for size distribution on an Experion Automated Electrophoresis System. Sequencing libraries were pooled and sequenced on a NextSeq 500 Desktop Sequencer (Illumina) using a NextSeq 500 High Output Kit v2 with 75 bp single-end reads. Raw sequencing data was demuxed using bcl2fastq2 Conversion Software 2.17 before alignment. Quality filtered reads were aligned to the reference human genome (hg19) using TopHat2 [72]. Transcript and gene level quantifications (in FPKM) were estimated using Cufflinks [73].

### Identification of differentially expressed genes (DEG)

Differentially expressed genes (DEGs) were identified using Cuffdiff. Transcripts with at least 10 FPKM in any of the conditions (ERG+ or ERG-) were used for differential gene expression analysis. We found 526 DEGs with a *q*-value ≤ 0.05, among which 117 genes were differentially expressed in ERG+ LnTE3 cells compared to ERG- control cells by at least |Log_10_FC| ≥2. Gene ontology analysis was performed in DAVID GO [74] and Pathway analysis were performed sing Ingenuity Pathway Analysis (QIAGEN Bioinformatics, USA).

### Real-time PCR and western blotting

Total RNA was isolated using the mirVana miRNA Isolation Kit (Invitrogen, AM1560) following the manufacturer’s instructions. After RNA extraction, RNA samples were reverse-transcribed using High Capacity cDNA Reverse Transcription Kit (Applied Biosystems, 4368813). Real time quantifications of TMPRSS2-ERG fusion mRNA was performed with specific TaqMan gene expression assay (Assay ID: Hs03063375_ft). Real-time PCR data were normalized to the endogenous control β-actin. The relative fold changes of candidate genes were analyzed by using 2^–ΔΔCT^ method.

Protein extraction and immunoblot analysis were performed using the standard protocol. In brief, cells were lysed in RIPA buffer supplemented with protease/phosphatase inhibitors (Sigma, P5726 and S8820, respectively). Samples containing 10μg protein were electrophoresed on a 4–12% Tris-Glycine gel. The separated proteins were electro-transferred to a nitrocellulose membrane (Bio-Rad, 1620112) for western blot analysis. All primary antibodies were used at 1:1000 dilution. The band intensities representing different protein expression levels were quantitated with reference to Glyceraldehyde 3-phosphate dehydrogenase (GAPDH) control bands. The intensities of protein bands were quantitated using ImageJ Gel Analysis program.

### Cell cycle and cell proliferation analysis

LnTE3 cells treated with or without doxycycline for 24 h and washed with PBS followed by trypsinization, and resuspended as a single cell suspension in PBS. Cells were fixed with 70% ethanol at a density of 1 million/ml and stored at 4° C for at least overnight. Fixed cells were again washed with PBS, treated with 200 μg/ml RNase-A for 30 min at 37° C. These cells are stained with 50 μg/mL propidium iodide and incubated at 4° C for 10 min. Cell cycle distribution was studied with the help of BD LSR II (Becton-Dickinson & CO., USA) flow cytometer. Cell proliferation was performed using Promega’s Cell Titer Aqueous kit. Briefly, 1 × 10^4^ LnTE3 cells/well were seeded in a 96-well plate with and without dox and incubated for 24 h. Subsequently after ensuring proper cell adhesion, media was changed, with and without dox as per requirement. The absorbance at 490 nm was measured using a microplate reader (BMG labtech) from 2 to 5 days interval.

## SUPPLEMENTARY MATERIALS





## Abbreviations

RNA-seq: RNA sequencing
UTR: Untranslated Region
CaP: Prostate Cancer
ERG: V-Ets Avian Erythroblastosis Virus E26 Oncogene Homolog
AR: Androgen Receptor
TMPRSS2: Transmembrane Protease Serine2
IPA: Ingenuity Pathway Analysis.
