# Supplementary material for: Comparative RNA-seq analysis reveals dys-regulation of major canonical pathways in ERG-inducible LNCaP cell progression model of prostate cancer
Source: Oncotarget. 2019 Jul 2;10(42):4290–306. doi: 10.18632/oncotarget.27019 (PMC6611515; doi:10.18632/oncotarget.27019)
Supplement: Supplementary file 1 [file oncotarget-10-4290-s001.pdf]

Comparative RNA-seq analysis reveals dys-regulation of major canonical pathways in ERG-inducible LNCaP cell progression model of prostate cancer

SUPPLEMENTARY MATERIALS

Supplementary Table 1: Data analysis of RNA-seq in ERG+ and ERG– LnTE3 cell lines

LNCaP cell	Sample ID	Total reads	Reads mapped	% of reads mapped	Multiple alignments	% Multiple alignment
ERG+	RB3	161,69,619	145,06,018	89.71	2,80,384	1.93
	RB4	180,28,229	159,82,569	88.65	3,20,275	2.00
	RB5	231,01,934	205,20,329	88.83	4,26,914	2.08
ERG–	RB8	95,00,037	85,92,376	90.45	1,86,445	2.17
	RB10	224,85,729	205,19,608	91.26	4,34,975	2.12

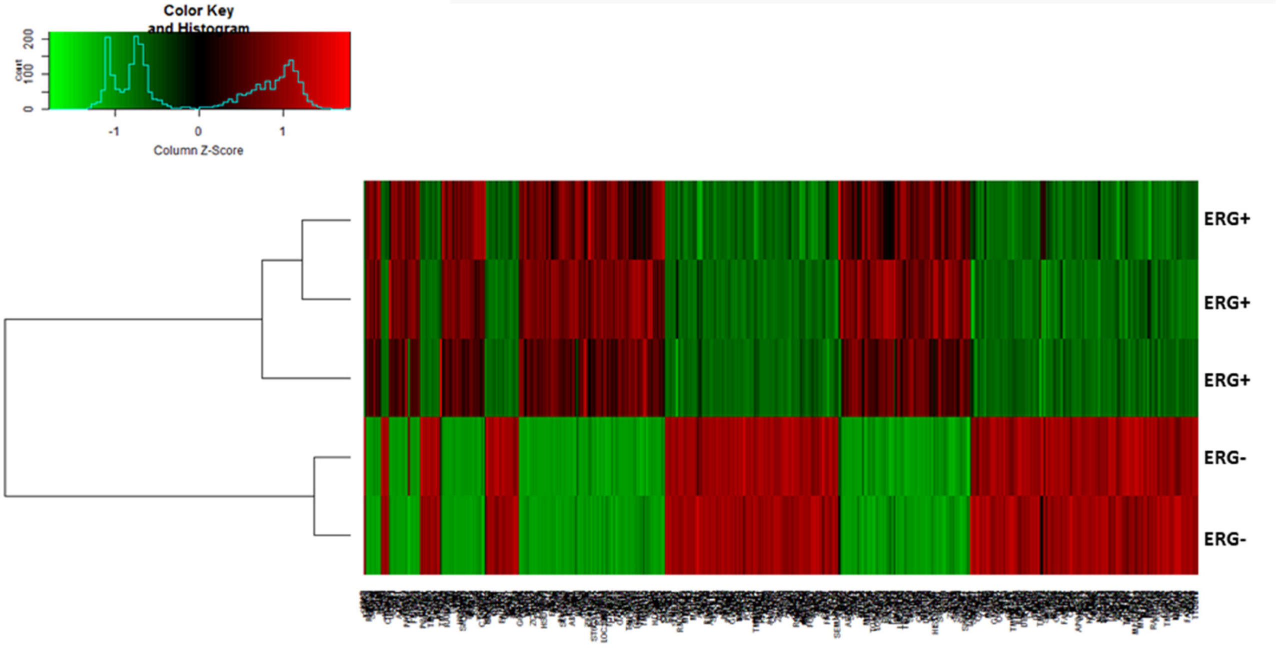

Supplementary Figure 1: Expression profiles and hierarchical clustering of ERG-modulated transcripts. Red represents increased expression; green, reduced expression.

Supplementary Data 1: Summary of ERG modulated transcripts: List of significant genes that were up regulated or down regulated with increased ERG expression in LnTE3 cells. See Supplementary Data 1
